# Decision-making difficulties mediate the association between poor emotion regulation and eating disorder symptoms in adolescence

**DOI:** 10.1017/S003329172200037X

**Published:** 2023-06

**Authors:** Marta Francesconi, Eirini Flouri, Amy Harrison

**Affiliations:** Department of Psychology and Human Development, Institute of Education, University College London, 25 Woburn Square, London WC1H 0AA, UK

**Keywords:** Cambridge Gambling Task, decision-making, eating pathology, emotional dysregulation, Millennium Cohort Study, self-regulation

## Abstract

**Background:**

The emergence of eating problems during childhood increases the risk for eating disorders (EDs) during young adulthood. Previous studies highlight a relationship between poor self-regulation and onset of eating pathology. In this study, we investigated whether this association is mediated by decision-making difficulties.

**Methods:**

To test this hypothesis, we used data from the Millennium Cohort Study. Decision-making performance was assessed with the Cambridge Gambling Task at age 11. Principal components analysis was used to derive an index of ED symptoms at age 14. The trajectories of scores of two subscales of the Child Social Behaviour Questionnaire, Independence and Self-Regulation (ISR) and Emotional Dysregulation (EmotDy), were modelled from ages 3 to 7 years in a latent growth curve analysis. The individual predicted values of the intercept (set at baseline, 3 years) and the slope (rate of annual change) were then used in the mediation analysis.

**Results:**

In our sample of 11 303 individuals, there was evidence for mediation by three measures of decision-making at age 11 (poor quality of decision-making, delay aversion and low risk-adjustment) in the association between EmotDy across ages 3–7 and ED symptoms at age 14 even after the adjustment for relevant covariates. We found no evidence of association between ISR and ED symptoms.

**Conclusion:**

Our findings suggest that emotion regulation processes during childhood may be relevant for the future onset of ED symptoms via their association with decision-making skills. These findings, obtained from a large, representative, sample, shed light on the relationship between self-regulation, decision-making and symptoms of EDs.

## Introduction

Eating disorders (EDs) are severe mental illnesses known to have physical, psychological and cognitive impacts (Smink, van Hoeken, & Hoek, 2013; Smith, Xiao, & Bechara, 2012). Individuals with EDs often report serious medical morbidity and psychiatric comorbidity (Smith et al., 2012; Steinhausen, 2009) and the National Health Service (NHS) has recently estimated that costs for EDs across the UK are likely to be more than £6.8–8 billion per year (Beat, 2015; Demmler, Brophy, Marchant, John, & Tan, 2020). ED symptomatology typically begins during early adolescence (Kotler, Cohen, Davies, Pine, & Walsh, 2001; Zipfel, Giel, Bulik, Hay, & Schmidt, 2015) and having problems with eating, weight and shape during childhood increases the risk for EDs developing in young adulthood 7.3 times (Kotler et al., 2001). Therefore, there is an urgent need to understand what early factors are involved in the onset of eating problems.

Self-regulation represents the capacity to adjust cognitive, emotional and behavioural reactions to reach a desired outcome. It includes being able to resist highly emotional reactions to upsetting situations, to calm yourself down when you get upset, to adjust to a change in expectations, and to handle frustration without an outburst. The strategies that are used to regulate these responses may be adaptive, such as reappraisal, problem-solving and acceptance or maladaptive, such as suppression, avoidance and rumination (Aldao, Nolen-Hoeksema, & Schweizer, 2010). Self-regulation has been identified as a potential risk factor for EDs (Donofry, Roecklein, Wildes, Miller, & Erickson, 2016; Mallorquí-Bagué et al., 2018; Prefit, Cândea, & Szentagotai-Tătar, 2019). Recent evidence shows that self-regulation processes play a crucial role in many developmental outcomes and may contribute to the maintenance of EDs (Treasure & Schmidt, 2013). In particular, it has been found that patients with EDs show poorer behavioural control and difficulty in their overall ability to regulate emotions (Johnson & Wardle, 2005; Ruscitti, Rufino, Goodwin, & Wagner, 2016).

One explanation for such findings may be that ED symptoms, like restricting, bingeing and purging, may function as coping mechanisms to unpleasant emotional states (Burns, Fischer, Jackson, & Harding, 2012; Overton, Selway, Strongman, & Houston, 2005).

The ability to regulate emotional and behavioural responses starts developing during early childhood and its stability over time is well established (Edossa, Schroeders, Weinert, & Artelt, 2018; Raffaelli, Crockett, & Shen, 2005). Given its developmental relevance, self-regulation has also been studied in the context of executive functioning skills, including attentional control, cognitive inhibition, inhibitory control, working memory and decision-making (Dohle, Diel, & Hofmann, 2018; Kittel, Schmidt, & Hilbert, 2017), but also decision-making under risk (Martin & Delgado, 2011). Decision-making skills represent the ability to select between two or more alternatives to reach the best outcome in the shortest time. In a recent study, the authors found attenuated blood-oxygen-level-dependent (BOLD) signals in the striatum, a brain structure previously linked with reward-related processing, in those showing strengths in emotion regulation (Martin & Delgado, 2011). In turn, reward-related processing, risk-taking and decision-making under risk in general are related to EDs (Harrison, O'Brien, Lopez, & Treasure, 2010; Smith et al., 2012). For example, we recently found in a large population sample that the risk of developing ED symptoms was higher in those who showed higher risk-taking and lower in those with better decision-making (Francesconi, Flouri, & Harrison, 2020).

However, what we did not investigate, and is still unexplored, is the possible role played by self-regulation processes in the association between decision-making and ED symptoms. Increasing knowledge on the association between self-regulation, decision-making and EDs will help understanding of the development of eating pathology. Moreover, it will provide evidence for planning targeted interventions, which are greatly needed especially in young individuals. Thus, this study aims to be the first to test the hypothesis that decision-making skills (measured with a gambling task) in late childhood mediate the association between self-regulation abilities in early childhood and ED symptoms in early adolescence. We hypothesised that decision-making ability could play a mediation role, given its strong association with both ED pathology and self-regulation. To achieve this, we examined associations between the trajectories of two domains of children's self-regulation, (a) independence and self-regulation and (b) emotional dysregulation across ages 3–7 with decision-making abilities at age 11, in turn associated with ED symptoms at age 14 while adjusting for pubertal status, maternal psychological distress, family poverty, IQ and other relevant covariates. The ED symptoms that we considered in our study describe subtle, prodromal signs of eating pathology. Exploring the prodromal phase which precedes the onset of ED, rather than a full-blown syndrome, may provide helpful information to structure earlier intervention and thus achieve better outcomes.

## Methods

### Study sample

The Millennium Cohort Study (MCS) has been following the lives of 19 244 young people born across England, Scotland, Wales and Northern Ireland in 2000–2002 over a series of six sweeps. The study began with an original sample of 18 818 cohort members (Plewis, Calderwood, Hawkes, Hughes, & Joshi, 2007). The main data collection methods used during the study have included questionnaires, cognitive assessments and interviewer-administered physical measurements. The MCS sample was designed to provide a proper representation of the total population. In order to do so, certain subgroups of the population were intentionally over-sampled, namely children living in disadvantaged areas, children of ethnic minority backgrounds and children growing up in the three smaller nations of the UK. The disproportionate representation of these groups ensures that typically hard to reach populations are adequately represented and that sample sizes are sufficient for the analysis of ethnic minorities, those from disadvantaged backgrounds and children within each of the UK nations. In this study, we used data from sweep 2 (age 3), when self-regulation was first measured in the MCS, to sweep 6 (age 14), the last available sweep of data, and when eating, dieting and body image questions were asked in MCS. Our analytic sample included singletons and first-born twins or triplets with available information on eating, dieting or body image questions at age 14 and with available data on the Cambridge Gambling Task (CGT) at either age it was measured in MCS, i.e. at 11 or 14 years (*n* = 11 303).

### Measures

#### Decision-making under risky conditions

The CGT was developed to assess decision-making and risk-taking behaviour outside a learning context (Deakin, Aitken, Robbins, & Sahakian, 2004; Rogers et al., 1999). It is a subtest of the widely used and well-validated Cambridge Neuropsychological Test Automated Battery (Robbins et al., 1998). Participants are presented with a row of 10 boxes across the top of the screen, of which some are red and some are blue, and are told that a token is hidden behind one of them. They have to choose (a) which colour of box they believe the token is hidden behind (red or blue), and (b) the number of points they want to gamble. The proportion of red to blue boxes (box ratio) varies during the task pseudo-randomly to assess the influence of statistical risk (probability) on decision-making. The five CGT measures of decision-making that are used in this study include (1) the mean proportion of points bet on trials where the most probable colour was selected (risk-taking); (2) the mean proportion of trials where the most probable colour was selected (quality of decision-making); (3) the mean reaction time (in milliseconds) for making a selection (deliberation time); (4) the tendency to stake higher bets on favourable compared to unfavourable trials (risk adjustment); and (5) the time participants are prepared to wait in order to place a higher or lower bet (delay aversion). A sixth CGT measure, overall proportion bet (i.e. the mean proportion of points gambled across all trials), was excluded from our analysis in view of its very high correlation (>0.90) with risk-taking.

#### Eating disorder (ED) symptoms

In the MCS, a series of questions were asked at age 14 to assess eating and dieting attitudes and behaviours. These questions measured: body dissatisfaction (whether the participant reported a perception of their body as too overweight or not); intention to lose weight (the presence of a strong desire to weight); dietary restriction (whether the participant had ever actively eaten less to influence their shape/weight) and excessive exercise (whether the participant had ever exercised in a driven way in order to influence weight and shape). We also included in our analysis an objective measure of underweight and overweight based on the most widely-used reference panel, the UK90, which is sensitive to gender and age and developed for the British population. Cut-offs in our sample were based on the age of the cohort member at the time of interview. The underweight cut-off point was the second centile and the overweight cut-off point was the 85th centile, as suggested by the UK90 (Cole, Freeman, & Preece, 1995). Online Supplementary Table S3 reports the list of questions used to assess ED symptoms in this study.

#### Self-regulation

During the self-completion module of the parent-interview at ages 3, 5 and 7, parents completed 10 items from the Child Social Behaviour Questionnaire (CSBQ) (Hogan, Scott, & Bauer, 1992). The main respondent completed two CSBQ subscales: the Independence and Self-Regulation (ISR) subscale which contains five items related to cognitive self-regulation (e.g. ‘persists in the face of difficult tasks’) and the Emotional Dysregulation (EmotDy) subscale which contains five items related to emotional self-regulation (e.g. ‘is easily frustrated’). Responses were on a three-point scale (not true, somewhat true and certainly true). Higher scores indicate more independence on the ISR subscale and more emotional dysregulation on the EmotDy subscale. All items were averaged, creating a total score ranging from 1 to 3. The full list of items can be found in online Supplementary Table S3.

#### Covariates

We controlled for a number of covariates known to be associated with exposure and outcome, including gender, ethnicity (according to the UK census groups of white, black, Indian, Pakistani/Bangladeshi, mixed or other), family poverty when the cohort child was aged 3 (below the poverty line or not), maternal mental health, assessed with the six-item Kessler scale of psychological distress (Kessler et al., 2003) when the cohort child was aged 3, IQ, measured in the MCS at age 3 with the Naming Vocabulary subscale of the British Ability Scales (BAS) (Elliott, Smith, & McCulloch, 1996) and pubertal status at age 11 (breast growth or menstruation or hair on body for females, and voice change or facial hair or hair on body for males).

### Statistical analysis

We performed a mediation analysis to test the association between the trajectories of self-regulation (measured at ages 3, 5 and 7) and ED symptoms at age 14, and the intervening role of decision-making ability in this association. All analyses were performed in STATA 16.0. In all models, the MCS sampling stratum was controlled to account for the disproportionate stratification of the MCS survey design. To better understand the participants’ ED symptoms, and to simplify results, we used principal components analysis (PCA) to combine into a single index the five items assessing eating and dieting attitudes and behaviour. We then fitted latent growth curve models to estimate the longitudinal trajectories of self-regulation from ages 3 to 7 years for both ISR and EmotDy subscales separately. The individual predicted values of the intercept (set at baseline at age 3 years) and the slope (rate of annual change) were then used in the mediation models. Using the predict command in STATA, we generated predictions for the out-of-sample cases, i.e. the cases that were not used in the original estimation. This command uses the maximum likelihood with missing values (MLMV) estimation for those who had data on at least one time-point and single imputation for those missing information on all time-points. In this way, both the intercepts of ISR and EmotDy and their slopes were estimated for our whole analytic sample (*n* = 11 303). The individual predicted values of the intercepts and the slopes were then saved and used in regression models testing for mediation. Missingness ranged between 0.1% (ethnicity) and 28.7% (risk adjustment at age 11), and, to handle it, we used multiple imputation by chained equations (MICE) (20 imputed datasets) (Royston & White, 2011). To predict missing data, we used all variables selected for analysis models. During the imputation process, the MCS sampling stratum was controlled to account for the disproportionate stratification of the MCS sample. Mediation models were fitted using seemingly unrelated regression analysis.

## Results

### Principal components analysis (PCA)

The PCA confirmed the presence of a general underlying factor for the assessment of ED risk symptoms. The Kaiser-Meyer-Olkin (KMO) test value was 0.75 indicating that the sampling was adequate. The component extracted showed positive loadings of roughly equal size on all variables which can be interpreted as an overall indicator of ED risk symptoms. The component accounted for 47% of the total variance. The loadings of the items on the underlying factor were as follows: body dissatisfaction 0.24, intention to lose weight 0.53, dietary restriction 0.50, excessive exercise 0.47 and the UK90 cut-offs 0.40.

### Descriptive analysis

A total of 11 303 participants had at least one available measure of ED symptoms at age 14 and a valid measure of CGT at age 11. Multicollinearity among CGT measures was assessed by inspection of the variance inflation factor (VIF) values. The risk-taking and the overall proportion bet variables were very highly intercorrelated as discussed (*r* > 0.90, *p* < 0.001). They showed a VIF of 13.82, and we therefore excluded the overall proportion bet score from further analyses. Table 1 shows the descriptive statistics for the study variables including means and proportions for exposures, outcomes and covariates. The majority of our sample was not underweight or overweight according to the UK90 reference panel. A small proportion of our sample reported a perception of their body as very overweight whereas almost half of our sample reported dietary restraint and dieting behaviours. Finally, the majority of our analytic sample reported the use of exercise to influence body weight. Tables 2 and 3 show the correlation among exposures, outcomes and mediators. Correlations were generally low to moderate and the EmotDy subscale had higher correlations with both the CGT measures and the ED risk symptoms compared to the ISR subscale. EmotDy and ISR subscales were inversely related at all time-points, albeit weakly (−0.09 at age 3, −0.25 at age 5 and −0.32 at age 7).

**Table 1.** Descriptive statistics in the analytic sample (*n* = 11 303)

Continuous variables		
	*N*	*M* (s.d.)
CGT risk-taking, age 11	10 301	0.52 (0.16)
CGT quality of decision-making, age 11	10 302	0.80 (0.16)
CGT deliberation time, age 11	10 302	3329.90 (1328.43)
CGT risk adjustment, age 11	8052	1.02 (0.82)
CGT delay aversion, age 11	9201	0.33 (0.19)
CSBQ independence self-regulation, age 3	9943	2.46 (0.34)
CSBQ independence self-regulation, age 5	10 368	2.53 (0.34)
CSBQ independence self-regulation, age 7	10 173	2.51 (0.36)
CSBQ emotional dysregulation, age 3	9945	1.86 (0.45)
CSBQ emotional dysregulation, age 5	10 368	1.70 (0.45)
CSBQ emotional dysregulation, age 7	10 175	1.70 (0.45)
IQ, age 3	9836	49.96 (11.52)
Maternal psychological distress, age 3	9146	3.19 (3.65)

Categorical variables		
	*N*	%
Body dissatisfaction (*perception of body as very overweight*)	510	4.61
Dieting behaviours (*trying to lose weight*)	4659	42.05
Dietary restraint (*has ever eaten less to lose weight*)	4935	44.62
Excessive exercise (*use of exercise to influence body weight*)	6687	60.36
Underweight cut-off point for child's age and sex – significant underweight	169	1.56
Overweight cut-off point for child's age and sex – significant overweight	3751	34.52
Pubertal status (female), age 11	5614	59.13
Pubertal status (male), age 11	4686	49.15
Gender – female	5671	50.17
Below poverty line, age 3	3122	30.20
*Ethnicity*		
White	9250	81.92
Mixed	323	2.86
Indian	308	2.73
Pakistani and Bangladeshi	867	7.68
Black or Black British	363	3.21
Other ethnic group	180	1.59

CGT, Cambridge Gambling Task; CSBQ, Child Social Behaviour Questionnaire.

**Table 2.** Pearson's correlations of the main study variables in the analytic sample (*n* = 11 303)

	ED risk symptoms	CSBQ-ISR, age 3	CSBQ-ISR, age 5	CSBQ-ISR, age 7	CGT risk-taking, age 11	CGT quality of decision-making, age 11	CGT deliberation time, age 11	CGT risk adjustment, age 11	CGT delay aversion, age 11
ED risk symptoms	1								
CSBQ-ISR, age 3	0.00	1							
CSBQ-ISR, age 5	0.01	0.36**	1						
CSBQ-ISR, age 7	0.01	0.30**	0.48**	1					
CGT risk-taking, age 11	−0.02*	−0.05**	−0.07**	−0.08**	1				
CGT quality of decision-making, age 11	−0.04**	0.01	0.02**	0.05**	0.09**	1			
CGT deliberation time, age 11	0.02*	−0.00	−0.01	−0.04**	−0.05**	−0.21**	1		
CGT risk adjustment, age 11	−0.04**	0.00	0.03**	0.03**	−0.36**	0.08**	−0.05**	1	
CGT delay aversion, age 11	0.01	−0.01	−0.05**	−0.07**	0.12**	−0.12**	−0.11**	−0.17**	1

Note: ED, eating disorder; CGT, Cambridge Gambling Task; CSBQ, Child Social Behaviour Questionnaire; ISR, Independence Self-Regulation.

*p<0.05; **p<0.01.

**Table 3.** Pearson's correlations of the main study variables in the analytic sample (*n* = 11 303)

	ED risk symptoms	CSBQ-EMotDy, age 3	CSBQ-EMotDy, age 5	CSBQ-EMotDy, age 7	CGT risk-taking, age 11	CGT quality of decision-making, age 11	CGT deliberation time, age 11	CGT risk adjustment, age 11	CGT delay aversion, age 11
ED risk symptoms	1								
CSBQ-EMotDy, age 3	0.02*	1							
CSBQ-EMotDy, age 5	0.04**	0.52**	1						
CSBQ-EMotDy, age 7	0.03**	0.47**	0.64**	1					
CGT risk-taking, age 11	−0.02*	0.06**	0.08**	0.08**	1				
CGT quality of decision-making, age 11	−0.04**	−0.06**	−0.06**	−0.08**	0.09**	1			
CGT deliberation time, age 11	0.02*	0.02*	0.03**	0.02**	−0.05**	−0.21**	1		
CGT risk adjustment, age 11	−0.04**	−0.06**	−0.05**	−0.06**	−0.36**	0.08**	−0.05**	1	
CGT delay aversion, age 11	0.01	0.06**	0.06**	0.08**	0.12**	−0.12**	−0.11**	−0.17**	1

Note: ED, eating disorder; CGT, Cambridge Gambling Task; CSBQ, Child Social Behaviour Questionnaire; EMotDy, Emotional Dysregulation.

*p<0.05; **p<0.01.

### Mediation models

We tested the effect of the intercept and the slope of EmotDy and ISR on ED risk symptoms via the CGT measures. We found no association between the intercept and the slope of ISR and ED symptoms (Table 4). However, we found an association between both the intercept and the slope of EmotDy and ED symptoms. This association was mediated by three out of the five CGT measures, as shown in Table 5. Quality of decision-making mediated the association between both the intercept and the slope of EmotDy and ED symptoms whereas risk adjustment and delay aversion mediated the association between only the intercept of EmotDy and ED symptoms. These results were robust to adjustment for covariates. Tables 4 and 5 present the results (direct, indirect and total effects) of our mediation models after adjustment in the imputed cases. We obtained the same results in the complete cases analysis, as displayed in online Supplementary Tables S1 and S2.

**Table 4.** Mediation (by CGT) models of independence-self regulation and ED risk symptoms

Direct and indirect paths	Imputed cases (*n* = 11 303)		
	*b*	s.e.	95% CI
Risk-taking			
Slope of ISR → risk-taking	−0.10**	0.03	−0.16 to −0.04
Intercept of ISR → risk-taking	−0.02**	0.00	−0.04 to −0.00
Risk-taking → ED symptoms	0.20*	0.09	0.01–0.40
Slope of ISR → ED symptoms	−0.06	0.29	−0.63 to 0.51
Intercept of ISR → ED symptoms	−0.07	0.08	−0.24 to 0.10
Indirect effect with slope as predictor	−0.02	0.01	−0.04 to 0.00
Total effect with slope as predictor	−0.08	0.29	−0.66 to 0.49
Indirect effect with intercept as predictor	−0.00	0.00	−0.00 to 0.00
Total effect with intercept as predictor	−0.07	0.08	−0.25 to 0.09
Quality of decision-making			
Slope of ISR → quality of decision-making	0.13**	0.03	0.07–0.19
Intercept of ISR → quality of decision-making	0.01	0.01	−0.00 to 0.03
Quality of decision-making → ED symptoms	−0.36**	0.09	−0.55 to −0.18
Slope of ISR → ED symptoms	−0.03	0.29	−0.61 to 0.54
Intercept of ISR → ED symptoms	−0.07	0.08	−0.24 to 0.09
Indirect effect with slope as predictor	−0.05**	0.01	−0.08 to −0.01
Total effect with slope as predictor	−0.08	0.29	−0.66 to 0.49
Indirect effect with intercept as predictor	−0.00	0.00	−0.01 to 0.00
Total effect with intercept as predictor	0.07	0.08	−0.25 to 0.09
Deliberation time			
Slope of ISR → deliberation time	−1146.93**	252.24	−1640.65 to −651.42
Intercept of ISR → deliberation time	−119.93	78.16	−273.24 to 33.37
Deliberation time → ED symptoms	0.00	0.00	−7.50 to 0.00
Slope of ISR → ED symptoms	−0.06	0.29	−0.64 to 0.51
Intercept of ISR → ED symptoms	−0.07	0.08	−0.24 to 0.09
Indirect effect with slope as predictor	−0.01	0.01	−0.04 to 0.00
Total effect with slope as predictor	−0.08	0.29	−0.66 to 0.49
Indirect effect with intercept as predictor	−0.00	0.00	−0.00 to 0.00
Total effect with intercept as predictor	−0.07	0.08	−0.25 to 0.09
Risk adjustment			
Slope of ISR → risk adjustment	0.36*	0.17	0.01–0.70
Intercept of ISR → risk adjustment	0.07	0.05	−0.02 to 0.17
Risk adjustment → ED symptoms	−0.06*	0.02	−0.10 to −0.02
Slope of ISR → ED symptoms	−0.06	0.29	−0.64 to 0.51
Intercept of ISR → ED symptoms	−0.07	0.08	−0.24 to 0.09
Indirect effect with slope as predictor	−0.02	0.01	−0.04 to 0.00
Total effect with slope as predictor	−0.08	0.29	−0.66 to 0.49
Indirect effect with intercept as predictor	−0.00	0.00	−0.01 to 0.00
Total effect with intercept as predictor	−0.07	0.08	0.25–0.09
Delay aversion			
Slope of ISR → delay aversion	−0.20**	0.04	−0.28 to −0.12
Intercept of ISR → delay aversion	−0.02*	0.01	−0.05 to −0.00
Delay aversion → ED symptoms	0.20*	0.08	0.04–0.35
Slope of ISR → ED symptoms	−0.04	0.29	−0.62 to 0.53
Intercept of ISR → ED symptoms	−0.07	0.08	−0.24 to 0.10
Indirect effect with slope as predictor	−0.04*	0.01	−0.07 to −0.00
Total effect with slope as predictor	−0.08	0.29	−0.66 to 0.49
Indirect effect with intercept as predictor	−0.00	0.00	−0.01 to 0.00
Total effect with intercept as predictor	−0.07	0.08	0.25–0.09

*b*, Unstandardised regression coefficient; s. e., standard error; CI, confidence interval; ED, eating disorder; CGT, Cambridge Gambling Task; ISR, Independence Self-Regulation.

Adjusted for: pubertal status, gender, ethnicity, maternal psychological distress at age 3, family poverty at age 3, IQ at age 3.

*p < 0.05; **p < 0.01.

**Table 5.** Mediation (by CGT) models of emotional dysregulation and ED risk symptoms

Direct and indirect paths	Imputed cases (*n* = 11 303)		
	*b*	s.e.	95% CI
Risk-taking			
Slope of EmotDy → risk-taking	0.08**	0.02	0.03–0.13
Intercept of EmotDy → risk-taking	0.02**	0.00	0.01–0.03
Risk-taking → ED symptoms	0.18	0.09	−0.00 to 0.38
Slope of EmotDy → ED symptoms	0.50*	0.24	0.02–0.97
Intercept of EmotDy → ED symptoms	0.21**	0.05	0.10–0.32
Indirect effect with slope as predictor	0.01	0.00	−0.00 to 0.03
Total effect with slope as predictor	0.52*	0.24	0.04–0.99
Indirect effect with intercept as predictor	0.00	0.00	−0.00 to 0.01
Total effect with intercept as predictor	0.21**	0.05	0.11–0.32
Quality of decision-making			
Slope of EmotDy → quality of decision-making	−0.09**	0.02	−0.15 to −0.04
Intercept of EmotDy → quality of decision-making	−0.03**	0.00	−0.04 to −0.02
Quality of decision-making → ED symptoms	−0.34**	0.09	−0.53 to −0.16
Slope of EmotDy → ED symptoms	0.48*	0.24	0.01–0.96
Intercept of EmotDy → ED symptoms	0.20**	0.05	0.09–0.31
Indirect effect with slope as predictor	0.03*	0.01	0.00–0.05
Total effect with slope as predictor	0.52*	0.24	0.04–0.99
Indirect effect with intercept as predictor	0.01**	0.00	0.00–0.01
Total effect with intercept as predictor	0.21**	0.05	0.11–0.32
Deliberation time			
Slope of EmotDy → deliberation time	332.30	221.12	−101.51 to 766.12
Intercept of EmotDy → deliberation time	100.19*	48.68	4.72–195.65
Deliberation time → ED symptoms	0.00	0. 00	−8.36 to 0.00
Slope of EmotDy → ED symptoms	0.51*	0.24	0.04–0.99
Intercept of EmotDy → ED symptoms	0.21**	0.05	0.10–0.32
Indirect effect with slope as predictor	0.00	0.00	−0.00 to 0.01
Total effect with slope as predictor	0.52*	0.24	0.04–0.99
Indirect effect with intercept as predictor	0.00	0.00	−0.00 to 0.00
Total effect with intercept as predictor	0.21**	0.05	0.11–0.32
Risk adjustment			
Slope of EmotDy → risk adjustment	−0.32*	0.16	−0.64 to −0.00
Intercept of EmotDy → risk adjustment	−0.11**	0.03	−0.17 to −0.05
Risk adjustment → ED symptoms	−0.06**	0.02	−0.10 to −0.01
Slope of EmotDy → ED symptoms	0.50*	0.24	0.02–0.97
Intercept of EmotDy → ED symptoms	0.21**	0.05	0.10–0.32
Indirect effect with slope as predictor	0.01	0.01	−0.00 to 0.04
Total effect with slope as predictor	0.52*	0.24	0.04–0.99
Indirect effect with intercept as predictor	0.00*	0.00	−0.00 to 0.01
Total effect with intercept as predictor	0.21**	0.05	0.11–0.32
Delay aversion			
Slope of EmotDy → delay aversion	0.13**	0.03	0.07–0.20
Intercept of EmotDy → delay aversion	0.03**	0.00	0.02–0.05
Delay aversion → ED symptoms	0.18*	0.08	0.02–0.34
Slope of EmotDy → ED symptoms	0.49*	0.24	0.01–0.97
Intercept of EmotDy → ED symptoms	0.21**	0.05	0.10–0.32
Indirect effect with slope as predictor	0.02	0.01	−0.00 to 0.05
Total effect with slope as predictor	0.52*	0.24	0.04–0.99
Indirect effect with intercept as predictor	0.00*	0.00	−0.00 to 0.01
Total effect with intercept as predictor	0.21**	0.05	0.11–0.32

*b*, Unstandardised regression coefficient; s.e., standard error; CI, confidence interval; ED, eating disorder; CGT, Cambridge Gambling Task; EmotDy, Emotional Dysregulation.

Adjusted for: pubertal status, gender, ethnicity, maternal psychological distress at age 3, family poverty at age 3, IQ at age 3.

*p < 0.05; **p < 0.01.

## Discussion

The aim of the study was to test for the first time the mediator effect of decision-making abilities at age 11 (delay aversion, risk adjustment, risk-taking, quality of decision-making and deliberation time) on the longitudinal association between self-regulation across ages 3–7 (ISR and EmotDy) and ED symptoms measured at age 14. We found, in a large population sample of 11 303 individuals, that poor quality of decision-making, poor risk adjustment and delay aversion mediated the positive association between EmotDy and ED symptoms and this result was robust to adjustment for covariates.

With respect to the trajectories of ISR and EmotDy from age 3 to 7 that we estimated, our sample showed a small increase over time in ISR, and a small decline over time in EmotDy. These results support previous findings describing self-regulation as a stable ability (Edossa et al., 2018; Raffaelli et al., 2005). We found that both the intercept (set at age 3) and the slope (which measured the rate of change from age 3 to 7 years) of EmotDy were associated with ED symptoms at age 14 and this finding is in line with previous evidence that identified this specific construct of self-regulation as a risk factor for the development and maintenance of all forms of eating pathology (Mallorquí-Bagué et al., 2018; Prefit et al., 2019). It has been hypothesised that ED symptoms may be maladaptive strategies used to manage negative emotions, hence the expression of poor emotion regulation (Burns et al., 2012; Fairburn, 2008; Harrison, Sullivan, Tchanturia, & Treasure, 2009; Overton et al., 2005; Treasure & Schmidt, 2013). Following this hypothesis, dysfunctional eating behaviours such as vomiting, excessive exercise, eating restriction and binge eating would emerge to regulate unpleasant emotional states (Evers, Marijn Stok, & de Ridder, 2010; Harrison et al., 2009). This explanation indicates that the emotion regulation processes may have a central role in the aetiology of ED symptom development as is also suggested by our findings. In view of the evidence that emotion regulation strategies can be modified (Mallorquí-Bagué et al., 2018; Preyde, Watson, Remers, & Stuart, 2016), this in turn would suggest that a clear implication of these findings is the need for emotional skill interventions in early childhood. Mounting evidence supports the efficacy of early, comprehensive and developmentally based prevention programmes, such as The Incredible Years programmes, for reducing early risk factors related to several adverse outcomes in adolescence (Marcynyszyn, Maher, & Corwin, 2011; Webster-Stratton, Jamila Reid, & Stoolmiller, 2008). Our results indicate that such programmes may be used to target dysfunctional emotion regulation during childhood with the final aim of reducing the risk for ED symptoms during adolescence.

The longitudinal association between decision-making abilities and ED symptoms identified in this study also corroborates previous findings regarding the role of cognition in the development of EDs (Garrido & Subirá, 2013; Harrison et al., 2010; Kittel et al., 2017). Recent studies found that reduced functionality of the ventromedial prefrontal cortex was associated with worse decision-making performance in ED patients, suggesting a common substrate for impaired decision-making in EDs (Reiter, Heinze, Schlagenhauf, & Deserno, 2017).

Finally, our results show, for the first time, that the association between EmotDy during early childhood and the expression of ED symptoms during adolescence was mediated by three out of five measures of decision-making. In particular, we found that (i) higher EmotDy was associated with lower quality of decision-making and with lower risk adjustment in turn associated with higher levels of ED symptoms, and that (ii) higher EmotDy was associated with more delay aversion in turn associated with higher ED symptomatology. The ability to regulate emotion is an essential coping mechanism and its development precedes the development of higher cognitive functions such as decision-making (Gilmore et al., 2012; Martin & Delgado, 2011). In fact, from a neurodevelopmental perspective, the development of subcortical brain structures involved in the emotional processes, such as the amygdala, predates and may influence the development of cortical brain regions engaged with decision-making abilities such as the dorsolateral prefrontal cortex and the ventromedial prefrontal cortex (Delgado, Nearing, LeDoux, & Phelps, 2008; Gilmore et al., 2012). These cortical areas also showed reduced functionality in patients with EDs (Goodkind et al., 2015; Reiter et al., 2017; Voon et al., 2015). This interconnected neurobiological background could explain our findings.

Quality of decision-making mediated the association between both the intercept and the slope of EmotDy and ED symptoms, whereas risk adjustment and delay aversion were mediators of the association between the intercept, but not the slope, of EmotDy and ED symptoms. This result highlights the relevance of identifying early dysfunctional emotional regulation. We did not find any association between the ISR subscale and ED symptoms. This is in contrast with other studies which found impaired behavioural and cognitive regulation in individuals with EDs (Crino, Touyz, & Rieger, 2019; Johnson & Wardle, 2005). However, the behavioural and cognitive regulation domain was assessed in our study with a scale of five items, the ISR subscale, measuring the ability to focus and sustain attention and the ability or tendency to do things independently. The lack of specificity and the limited number of items may be an explanation for our null finding.

There are several important study limitations. First, given the observational design, causality cannot be inferred. Second, given the multidisciplinary nature of the MCS, we did not have a clinical interview for EDs available. However, it is also important to note that clinical diagnosis was not the focus of this work and that, as mentioned earlier, this study aimed to explore ED symptoms during early adolescence which as previously discussed are strongly related to high risk for EDs later in adolescence and early adulthood. Third, ED symptoms in the MCS were measured for the first time at age 14 so we could not control for them at age 11, when decision-making was first measured. Fourth, self-regulation was measured in the MCS with two scales, ISR and EmotDy, of five items each. As described earlier, self-regulation is a complex construct that includes cognitive, emotional and behavioural processes. In our study, we were not able to cover this complexity fully. However, we explored several important aspects of children's self-regulation. Finally, our sample showed different amounts of missing data across the CGT outcomes. There are two reasons that may explain this. First, in MCS, the CGT derived measures only included data when the most likely outcome was chosen, therefore some outcome measures do not include the rest of trials. Second, our analytic sample included singletons and first-born twins or triplets having at least one available CGT outcome and that answered to at least one eating-related question. This can explain the heterogeneous missingness rate across CGT outcomes. However, our sample selection strategy was chosen in order to be neither too conservative nor too liberal and we used a strong and reliable imputation strategy that relies on available data to replace missing data (MICE).

These limitations notwithstanding, our findings suggest that emotional dysregulation during early childhood is related to the emergence of ED symptoms during adolescence via suboptimal decision-making. These findings, obtained from a large, general population sample, provide an additional step towards understanding the relationship between self-regulation processes and symptoms of eating pathology.

## Data Availability

We used data from Millennium Cohort Study, which are publicly available and can be downloaded from the UK data service website (https://ukdataservice.ac.uk/).

## Supplementary material

For supplementary material accompanying this paper visit https://doi.org/10.1017/S003329172200037X. click here to view supplementary material
