# Supplementary material for: Decision-making difficulties mediate the association between poor emotion regulation and eating disorder symptoms in adolescence
Source: Psychol Med. 2022 Mar 1;53(8):3701–10. doi: 10.1017/S003329172200037X (PMC10277753; doi:10.1017/S003329172200037X)
Supplement: Supplementary file 1 [file S003329172200037Xsup001.docx]

| Table S1. Mediation (by CGT) Models of Independence-Self regulation and ED Risk Symptoms | | | |
| --- | --- | --- | --- |
| Direct and Indirect Paths | **Complete Cases** | | |
|  |  | | |
|  | **b** | **SE** | **95% CI** |
| Risk Taking (n=6,855) | | | |
| Slope of ISR 🡪 Risk Taking | -0.10**^**^** | 0.03 | -0.18 - -0.03 |
| Intercept of ISR 🡪 Risk Taking | -0.03**^**^** | 0.01 | -0.05 - -0.00 |
| Risk Taking 🡪 ED Symptoms | 0.26**^*^** | 0.11 | 0.04 - 0.48 |
| Slope of ISR 🡪 ED Symptoms | -0.32 | 0.35 | -1.01 - 0.35 |
| Intercept of ISR 🡪 ED Symptoms | -0.04 | 0.10 | -0.25 - 0.15 |
| Indirect Effect with Slope as predictor | -0.02 | 0.01 | -0.05-0.00 |
| Total Effect with Slope as predictor | -0.35 | 0.35 | -1.04-0.32 |
| Indirect Effect with Intercept as predictor | -0.00 | 0.00 | -0.01-0.00 |
| Total Effect with Intercept as predictor | -0.05 | 0.10 | -0.26-0.15 |
| Quality of decision making (n=6,856) | | | |
| Slope of ISR 🡪 Quality of decision making | 0.14**^**^** | 0.03 | 0.06 - 0.21 |
| Intercept of ISR 🡪 Quality of decision making | 0.00 | 0.01 | -0.01 - 0.03 |
| Quality of decision making 🡪 ED Symptoms | -0.42**^**^** | 0.10 | -0.63- -0.21 |
| Slope of ISR 🡪 ED Symptoms | -0.29 | 0.35 | -0.98 - 0.39 |
| Intercept of ISR 🡪 ED Symptoms | -0.05 | 0.10 | -0.26 - 0.15 |
| Indirect Effect with Slope as predictor | -0.05**^**^** | 0.02 | -0.10-0.01 |
| Total Effect with Slope as predictor | -0.35 | 0.35 | -1.04-0.33 |
| Indirect Effect with Intercept as predictor | -0.00 | 0.00 | -0.01-0.00 |
| Total Effect with Intercept as predictor | -0.05 | 0.10 | -0.26-0.14 |
| Deliberation Time (n=6,856) | | | |
| Slope of ISR 🡪 Deliberation Time | -1060.42**^**^** | 313.33 | -1674.55 - -446.292 |
| Intercept of ISR 🡪 Deliberation Time | -107.68 | 94.73 | -293.37- 77.99 |
| Deliberation Time 🡪 ED Symptoms | 0.00 | 0.00 | -0.00 -0.00 |
| Slope of ISR 🡪 ED Symptoms | -0.34 | 0.35 | -1.02-0.34 |
| Intercept of ISR 🡪 ED Symptoms | -0.05 | 0.10 | -0.26-0.15 |
| Indirect Effect with Slope as predictor | -0.01 | 0.01 | -0.04-0.01 |
| Total Effect with Slope as predictor | -0.35 | 0.35 | -1.04-0.33 |
| Indirect Effect with Intercept as predictor | -0.00 | 0.00 | -0.00-0.00 |
| Total Effect with Intercept as predictor | -0.05 | 0.10 | -0.26-0.14 |
| Risk Adjustment (n=5,441) | | | |
| Slope of ISR 🡪 Risk Adjustment | 0.39 | 0.21 | -0.02-0.82 |
| Intercept of ISR 🡪 Risk Adjustment | 0.03 | 0.06 | -0.08-0.16 |
| Risk Adjustment 🡪 ED Symptoms | -0.07**^**^** | 0.02 | -0.11- -0.02 |
| Slope of ISR 🡪 ED Symptoms | -0.66 | 0.39 | -1.43- 0.10 |
| Intercept of ISR 🡪 ED Symptoms | -0.05 | 0.11 | -0.28-0.18 |
| Indirect Effect with Slope as predictor | -0.02 | 0.01 | -0.06-0.00 |
| Total Effect with Slope as predictor | -0.69 | 0.39 | -1.46-0.08 |
| Indirect Effect with Intercept as predictor | -0.00 | 0.00 | -0.01-0.00 |
| Total Effect with Intercept as predictor | -0.05 | 0.11 | -0.28-0.17 |
| Delay Aversion (n=6,119) | | | |
| Slope of ISR 🡪 Delay Aversion | -0.19**^**^** | 0.04 | -0.29- -0.10 |
| Intercept of ISR 🡪 Delay Aversion | -0.01 | 0.01 | -0.04- 0.01 |
| Delay Aversion 🡪 ED Symptoms | 0.32**^**^** | 0.09 | 0.13-0.51 |
| Slope of ISR 🡪 ED Symptoms | -0.27 | 0.37 | -0.99-0.45 |
| Intercept of ISR 🡪 ED Symptoms | -0.04 | 0.11 | -0.26-0.17 |
| Indirect Effect with Slope as predictor | -0.06 | 0.02 | -0.11-0.01 |
| Total Effect with Slope as predictor | -0.33 | 0.37 | -1.06-0.39 |
| Indirect Effect with Intercept as predictor | -0.00 | 0.00 | -0.01-0.00 |
| Total Effect with Intercept as predictor | -0.05 | 0.11 | -0.27-0.16 |
| Note: b=Unstandardised regression coefficient; SE=Standard error; CI=Confidence interval; ED= Eating Disorder; CGT= Cambridge Gambling Task; ISR= Independence Self-Regulation  Adjusted for: pubertal status, gender, ethnicity, maternal psychological distress at age 3, family poverty at age 3, IQ at age 3  * p<0.05  **p<0.01 | | | |

| Table S2. Mediation (by CGT) Models of Emotional Dysregulation and ED Risk Symptoms | | | |
| --- | --- | --- | --- |
| Direct and Indirect Paths | **Complete Cases** | | |
|  |  | | |
|  | **b** | **SE** | **95% CI** |
| Risk Taking (n=6,855) | | | |
| Slope of EmotDy 🡪 Risk Taking | 0.09**^**^** | 0.03 | 0.03 - 0.15 |
| Intercept of EmotDy 🡪 Risk Taking | 0.03**^**^** | 0.00 | 0.02 - 0.05 |
| Risk Taking 🡪 ED Symptoms | 0.23**^*^** | 0.11 | 0.01 - 0.45 |
| Slope of EmotDy 🡪 ED Symptoms | 0.47 | 0.29 | -0.09 - 1.04 |
| Intercept of EmotDy 🡪 ED Symptoms | 0.26**^**^** | 0.06 | 0.12 - 0.39 |
| Indirect Effect with Slope as predictor | 0.02 | 0.01 | -0.00- 0.04 |
| Total Effect with Slope as predictor | 0.49 | 0.29 | -0.07-1.07 |
| Indirect Effect with Intercept as predictor | 0.00 | 0.00 | -0.00- 0.01 |
| Total Effect with Intercept as predictor | 0.26**^**^** | 0.06 | 0.13-0.39 |
| Quality of decision making (n=6,856) | | | |
| Slope of EmotDy 🡪 Quality of decision making | -0.08**^**^** | 0.03 | -0.15 - -0.02 |
| Intercept of EmotDy 🡪 Quality of decision making | -0.03**^**^** | 0.00 | -0.05- -0.02 |
| Quality of decision making 🡪 ED Symptoms | -0.39**^**^** | 0.10 | -0.60- -0.18 |
| Slope of EmotDy 🡪 ED Symptoms | 0.46 | 0.29 | -0.10 - 1.03 |
| Intercept of EmotDy 🡪 ED Symptoms | 0.25**^**^** | 0.06 | 0.12 - 0.38 |
| Indirect Effect with Slope as predictor | 0.03**^*^** | 0.01 | 0.00- 0.06 |
| Total Effect with Slope as predictor | 0.49 | 0.29 | -0.07-1.07 |
| Indirect Effect with Intercept as predictor | 0.01**^**^** | 0.00 | 0.00- 0.02 |
| Total Effect with Intercept as predictor | 0.26**^**^** | 0.06 | 0.13-0.39 |
| Deliberation Time (n=6,856) | | | |
| Slope of EmotDy 🡪 Deliberation Time | 235.46 | 261.64 | -277.35- 748.28 |
| Intercept of EmotDy 🡪 Deliberation Time | 74.33 | 48.68 | -42.10- 190.77 |
| Deliberation Time 🡪 ED Symptoms | 0.00 | 0. 00 | -0.00- 0.00 |
| Slope of EmotDy 🡪 ED Symptoms | 0.49 | 0.29 | -0.07 - 1.06 |
| Intercept of EmotDy 🡪 ED Symptoms | 0.26**^**^** | 0.06 | 0.13 - 0.39 |
| Indirect Effect with Slope as predictor | 0.00 | 0.00 | -0.00- 0.01 |
| Total Effect with Slope as predictor | 0.49**^*^** | 0.29 | -0.07-1.07 |
| Indirect Effect with Intercept as predictor | 0.00 | 0.00 | -0.00- 0.00 |
| Total Effect with Intercept as predictor | 0.26**^**^** | 0.06 | 0.13-0.39 |
| Risk Adjustment (n=5,441) | | | |
| Slope of EmotDy 🡪 Risk Adjustment | -0.42**^*^** | 0.18 | -0.78- -0.07 |
| Intercept of EmotDy 🡪 Risk Adjustment | -0.12**^**^** | 0.04 | -0.20- -0.04 |
| Risk Adjustment 🡪 ED Symptoms | -0.06**^**^** | 0.02 | -0.11- -0.01 |
| Slope of EmotDy 🡪 ED Symptoms | 0.72**^*^** | 0.32 | 0.08- 1.36 |
| Intercept of EmotDy 🡪 ED Symptoms | 0.32**^**^** | 0.07 | 0.17 - 0.46 |
| Indirect Effect with Slope as predictor | 0.02 | 0.01 | -0.00- 0.06 |
| Total Effect with Slope as predictor | 0.75**^*^** | 0.32 | 0.11-1.39 |
| Indirect Effect with Intercept as predictor | 0.00**^*^** | 0.00 | 0.00- 0.01 |
| Total Effect with Intercept as predictor | 0.33**^**^** | 0.07 | 0.18-0.47 |
| Delay Aversion (n=6,119) | | | |
| Slope of EmotDy 🡪 Delay Aversion | 0.15**^**^** | 0.04 | 0.07-0.23 |
| Intercept of EmotDy 🡪 Delay Aversion | 0.04**^**^** | 0.00 | 0.02-0.06 |
| Delay Aversion 🡪 ED Symptoms | 0.30**^**^** | 0.09 | 0.11-0.49 |
| Slope of EmotDy 🡪 ED Symptoms | 0.38 | 0.31 | -0.22 - 0.98 |
| Intercept of EmotDy 🡪 ED Symptoms | 0.23**^**^** | 0.07 | 0.09 - 0.37 |
| Indirect Effect with Slope as predictor | 0.04**^*^** | 0.01 | 0.00- 0.08 |
| Total Effect with Slope as predictor | 0.42 | 0.31 | -0.18-1.03 |
| Indirect Effect with Intercept as predictor | 0.01**^**^** | 0.00 | 0.00- 0.02 |
| Total Effect with Intercept as predictor | 0.24**^**^** | 0.07 | 0.11-0.38 |
| Note: b=Unstandardised regression coefficient; SE=Standard error; CI=Confidence interval; ED= Eating Disorder; CGT= Cambridge Gambling Task; EmotDy= Emotional Dysregulation  Adjusted for: pubertal status, gender, ethnicity, maternal psychological distress at age 3, family poverty at age 3, IQ at age 3  * p<0.05  **p<0.01 | | | |

**Table S3. Items measuring ED symptoms and Self-regulation construct.**

| **Eating Disorder Symptoms age 14** (yes/no) | **Self- regulation ages 3, 5 and 7**  (3-point scale. No true, somewhat true, certainly true) |
| --- | --- |
|  | *Independence scale* |
| Do you perceive your body as very overweight? | The child likes to work things out for self |
| Are you trying to lose weight? | The child does not need much help with tasks |
| Have you ever eaten less to lose weight | The child chooses activities on their own |
| Did you ever exercise to lose weight | The child persists in the face of difficult tasks |
| UK90 Underweight cut-off | The child moves to new activity after finishing task |
| UK90 Overweight cut-off | *Emotional dysregulation scale* |
|  | The child shows mood swings |
|  | The child gets over excited |
|  | The child is easily frustrated |
|  | The child gets over being upset quickly (reversed) |
|  | The child acts impulsively |
| **Note:** ED symptoms questions were administered to the study participant. Self-regulation was parent-reported. | |
